# A prognostic model of patients with ovarian mucinous adenocarcinoma: a population-based analysis

**DOI:** 10.1186/s13048-022-00958-6

**Published:** 2022-02-16

**Authors:** Li Yang, Jinfen Yu, Shuang Zhang, Yisi Shan, Yajun Li, Liugang Xu, Jinhu Zhang, Jianya Zhang

**Affiliations:** Zhangjiagang TCM Hospital Affiliated to Nanjing University of Chinese Medicine, 77Changan South Road, Zhangjiagang, 215600 Jiangsu Province China

**Keywords:** Ovarian mucinous adenocarcinoma, Overall survival, Cancer-specific survival, Nomogram

## Abstract

**Background:**

Ovarian mucinous carcinoma is a disease that requires unique treatment. But for a long time, guidelines for ovarian serous carcinoma have been used for the treatment of ovarian mucinous carcinoma. This study aimed to construct and validate nomograms for predicting the overall survival (OS) and cancer-specific survival (CSS) in patients with ovarian mucinous adenocarcinoma.

**Methods:**

In this study, patients initially diagnosed with ovarian mucinous adenocarcinoma from 2004 to 2015 were screened from the Surveillance, Epidemiology, and End Results (SEER) database, and divided into the training group and the validation group at a ratio of 7:3. Independent risk factors for OS and CSS were determined by multivariate Cox regression analysis, and nomograms were constructed and validated.

**Results:**

In this study, 1309 patients with ovarian mucinous adenocarcinoma were finally screened and randomly divided into 917 cases in the training group and 392 cases in the validation group according to a 7:3 ratio. Multivariate Cox regression analysis showed that the independent risk factors of OS were age, race, T_stage, N_stage, M_stage, grade, CA125, and chemotherapy. Independent risk factors of CSS were age, race, marital, T_stage, N_stage, M_stage, grade, CA125, and chemotherapy. According to the above results, the nomograms of OS and CSS in ovarian mucinous adenocarcinoma were constructed. In the training group, the C-index of the OS nomogram was 0.845 (95% CI: 0.821–0.869) and the C-index of the CSS nomogram was 0.862 (95%CI: 0.838–0.886). In the validation group, the C-index of the OS nomogram was 0.843 (95% CI: 0.810–0.876) and the C-index of the CSS nomogram was 0.841 (95%CI: 0.806–0.876). The calibration curve showed the consistency between the predicted results and the actual results, indicating the high accuracy of the nomogram.

**Conclusion:**

The nomogram provides 3-year and 5-year OS and CSS predictions for patients with ovarian mucinous adenocarcinoma, which helps clinicians predict the prognosis of patients and formulate appropriate treatment plans.

## Introduction

Ovarian cancer has the highest mortality rate among gynecologic malignancies [1]. Most ovarian cancer patients present with advanced disease at the time of initial diagnosis [2]. As one of the 10 most common cancers in women, the mortality rate of ovarian cancer is increasing [3]. There are an estimated 21,410 new cases of ovarian cancer and an estimated 13,770 deaths in the United States in 2021 [4]. Ovarian epithelial tumors are the most common histological type of ovarian cancer, which can be classified as benign, borderline, and malignant. The common types of ovarian epithelial tumors are serous tumors, mucinous tumors, ovarian endometrioid tumors, and clear cell tumors. Primary mucinous ovarian carcinoma is relatively rare among ovarian cancers [5]. A careful understanding of its biological characteristics shows that ovarian mucinous carcinoma is a disease that requires unique treatment. But for a long time, guidelines for ovarian serous carcinoma have been used for the treatment of ovarian mucinous carcinoma [6]. Therefore, it is significant to develop a predictive model to predict the prognosis and formulate individualized treatment plans for ovarian mucinous adenocarcinoma.

The nomogram predicts the probability of clinical events by integrating different determinants, which is in line with personalized medicine. Currently, the use of nomograms is also increasing [7]. Wang et al. [8] retrospectively analyzed 172 patients with primary ovarian signet cell carcinoma and constructed 1-year, 3-year, and 5-year OS nomograms, which were helpful for clinicians to assess the prognosis of patients. Wang et al. [9] screened 131,050 patients with epithelial ovarian cancer from the seed database and constructed nomograms of OS and CSS. Compared with the FIGO 2018 staging system, nomograms provided a better predictive effect.

In this study, we constructed and validated 3-year and 5-year OS and CSS nomograms in patients with ovarian mucinous adenocarcinoma based on the Surveillance, Epidemiology, and End Results (SEER) database. Nomograms help clinicians predict patient outcomes and formulate individualized treatment plans.

## Material and methods

### Patients selection

The Surveillance, Epidemiology, and End Results (SEER) database collects information on the demographics and clinical characteristics of cancer patients from cancer registries covering 47.9% of the U.S. population and publishes cancer incidence and mortality. It is updated once a year and is available to people for free [10]. We extracted relevant data from the SEER database for retrospective analysis by SEER*Stat 8.3.9 software (https://seer.cancer.gov/).

**Fig. 1:**
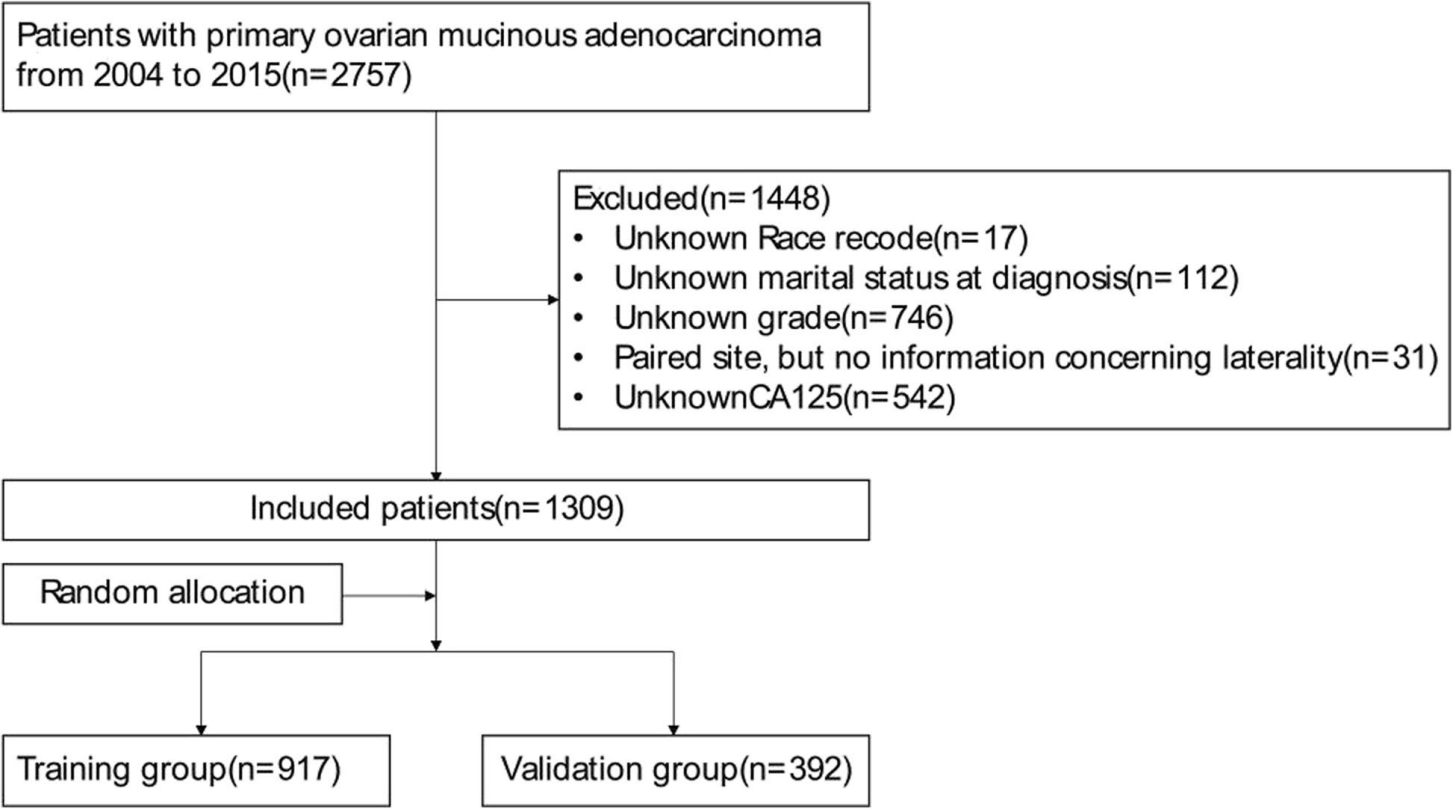
Flowchart for screening patients. A total of 2757 patients initially diagnosed with primary ovarian mucinous adenocarcinoma between 2004 and 2015 were screened through the database, of which 1309 were included in the analysis

We use the third edition of the International Classification of Diseases Oncology Special Edition (ICD-O-3) to screen for ovarian mucinous adenocarcinoma. ICD-O-3 includes anatomical codes and morphological codes. The inclusion criteria were the pathologically confirmed malignant mucinous adenocarcinoma as the first primary tumor and patients with anatomical codes (C56.9 (ovary)) between 2004 and 2015. The morphological codes were: 8470/3, 8471/3, 8472/3, 8473/3, 8480/3, and 8482/3. Then, we excluded Unknown Race recode(*n* = 17), Unknown marital status at diagnosis(*n* = 112), Unknown grade(*n* = 746), Paired site, but no information concerning laterality(*n* = 31), and Unknown CA125(*n* = 542). The flowchart is shown in Fig. 1.

### Predictors

We download the following data from the database: age, race, marital status, laterality, grade, AJCC TNM stage, CA125, surgery at the primary site, radiation therapy, chemotherapy, survival time, cause of death, and vital status. We included these data in subsequent analyses. This study focused on overall survival (OS) and cancer-specific survival (CSS). OS was defined as the time from diagnosis to death or last follow-up. CSS was defined as the time from diagnosis to death due to ovarian mucinous adenocarcinoma or last follow-up.

### Statistic methods

**Fig. 2:**
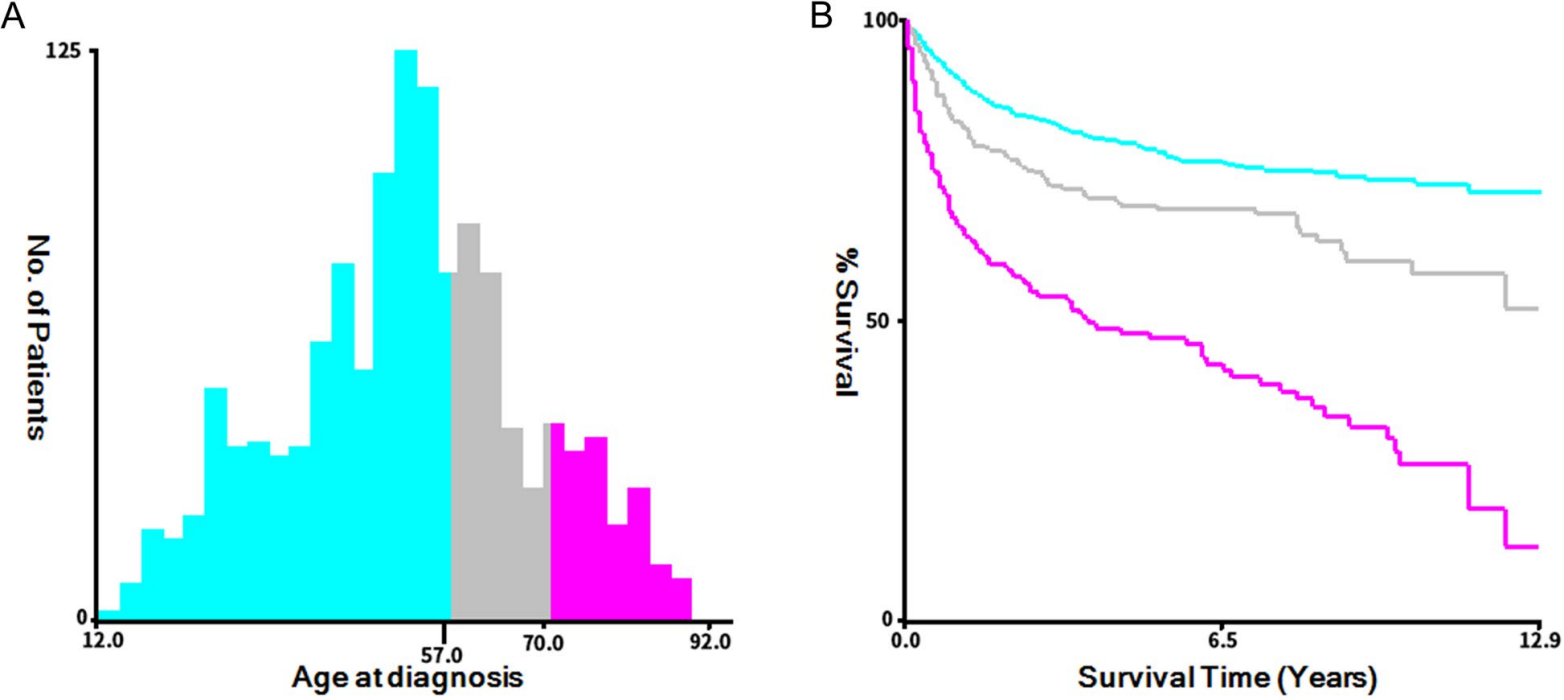
Identification of the best cut-off point of age (**A**, **B**) through X-tile software

In this study, 1309 patients with ovarian mucinous adenocarcinoma were finally screened and randomly divided into the training group and the validation group according to the ratio of 7:3 by R software (version 4.0.3). Finally, there were 917 cases in the training group and 392 cases in the validation group. We used X-tile software (version 3.6.1) to determine the optimal cut-off point for age (12–57 years, 58–70 years, 71–92 years) (Fig. 2).

We compared the variables screened in the training group and the validation group by Chi-square test. The *p*-value> 0.05 indicates that there is no statistical difference between the two groups, in other words, the two groups are comparable. Univariate Cox regression analysis was performed on the variables of the training group to screen out the risk factors related to OS and CSS in ovarian mucinous adenocarcinoma (*p* < 0.05). We performed multivariate Cox regression analysis of selected risk factors (*p* < 0.05). According to the risk factors screened by multivariate Cox regression analysis (*p* < 0.05), we could determine independent risk factors for OS and CSS. Prognostic nomograms of OS and CSS at 3 and 5 years were constructed according to their respective independent risk factors. We evaluated the discriminative ability of the model by the area under the ROC curve (AUC) and the C-index. Their value ranges and meanings were similar. Different ranges of AUC represent different meanings. The model had no predictive ability: AUC = 0.50; low accuracy: 0.50 < AUC ≤ 0.70; moderate accuracy: 0.70 < AUC ≤ 0.90; high accuracy: > 0.90. The C-index evaluated the probability that the predicted result was consistent with the actual observed result. It was used to evaluate the predictive ability of the model. The accuracy of the model was evaluated by the calibration curve, that is, it was used to compare the fit between the predicted situation and the actual situation. Taking the 45°diagonal in the figure as the reference line, the closer the actual probability line is to the reference line, the higher the accuracy of the model will be. The model was internally validated by the validation group, which verified the discriminative ability and accuracy of the model. All statistical analyses were performed with SPSS (version 25.0) and R software (version 4.0.3). A *p*-value < 0.05 was considered statistically significant.

## Results

### Demographic and clinical characteristics

A total of 1309 patients with ovarian mucinous adenocarcinoma were included in this study, including 917 (70.1%) in the training group and 392 (29.9%) in the validation group. Most patients were younger (12-57 years, 64.3%), white (80.0%), and married (48.7%) (Table 1). The majority of tumors were unilateral (88.5%), grade II (43.5%), AJCC stage T1 (74.1%), AJCC stage N0 (91.7%) and AJCC stage M0 (91.4%) (Table 1). Blood CA125 was mostly positive (67.6%) (Table 1). The vast majority of patients underwent surgery (99.2%), nearly half underwent chemotherapy (47.1%), and fewer patients underwent radiation therapy (0.9%) (Table 1).

**Table 1:** Demographics and Clinicopathologic Characteristics of the Training and Validation Groups in Ovarian Mucinous Adenocarcinoma Patients

Variables	Total		Training group		Validation group		*P* value
	n(%)		n(%)		n(%)		
Age							0.307
12–57	842	(64.3)	578	(63.0)	264	(67.3)	
58–70	291	(22.2)	213	(23.2)	78	(19.9)	
71–92	176	(13.4)	126	(13.7)	50	(12.8)	
Race							0.341
Black	101	(7.7)	70	(7.6)	31	(7.9)	
White	1047	(80.0)	742	(80.9)	305	(77.8)	
Other	161	(12.3)	105	(11.5)	56	(14.3)	
Marital							0.174
Divorced	142	(10.8)	94	(10.3)	48	(12.2)	
Married	638	(48.7)	461	(50.3)	177	(45.2)	
Separated	9	(0.7)	5	(0.5)	4	(1.0)	
Single	385	(29.4)	265	(28.9)	120	(30.6)	
Unmarried or Domestic Partner	4	(0.3)	1	(0.1)	3	(0.8)	
Widowed	131	(10.0)	91	(9.9)	40	(10.2)	
Laterality							0.425
unilateral	1158	(88.5)	807	(88.0)	351	(89.5)	
bilateral	151	(11.5)	110	(12.0)	41	(10.5)	
Grade							0.321
I	511	(39.0)	349	(38.1)	162	(41.3)	
II	569	(43.5)	405	(44.2)	164	(41.8)	
III	184	(14.1)	132	(14.4)	52	(13.3)	
IV	45	(3.4)	31	(3.4)	14	(3.6)	
T stage							0.546
T1	970	(74.1)	684	(74.6)	286	(73.0)	
T2	96	(7.3)	66	(7.2)	30	(7.7)	
T3	242	(18.5)	166	(18.1)	76	(19.4)	
TX	1	(0.1)	1	(0.1)	0	(0.0)	
N stage							0.891
N0	1201	(91.7)	842	(91.8)	359	(91.6)	
N1	61	(4.7)	42	(4.6)	19	(4.8)	
NX	47	(3.6)	33	(3.6)	14	(3.6)	
M stage							0.142
M0	1197	(91.4)	832	(90.7)	365	(93.1)	
M1	99	(7.6)	72	(7.9)	27	(6.9)	
MX	13	(1.0)	13	(1.4)	0	(0.0)	
CA125							0.645
Positive	885	(67.6)	612	(66.7)	273	(69.6)	
Negative	420	(32.1)	302	(32.9)	118	(30.1)	
Borderline	4	(0.3)	3	(0.3)	1	(0.3)	
Surgery							0.297
No	10	(0.8)	5	(0.5)	5	(1.3)	
Yes	1299	(99.2)	912	(99.5)	387	(98.7)	
Radiation							0.227
Yes	12	(0.9)	6	(0.7)	6	(1.5)	
None/Unknown	1297	(99.1)	911	(99.3)	386	(98.5)	
Chemotherapy							0.584
Yes	616	(47.1)	427	(46.6)	189	(48.2)	
No/Unknown	693	(52.9)	490	(53.4)	203	(51.8)	

### Independent risk factors for OS and CSS in patients with ovarian mucinous adenocarcinoma

Through univariate and multivariate Cox regression analysis on the training group data, the independent risk factors of OS and CSS were screened out. Univariate Cox regression analysis showed that they (age, race, marital, laterality, grade, T_stage, N_stage, M_stage, CA125, surgery, radiation, chemotherapy) were related to OS and CSS (*P* < 0.05) (Tables 2 and  3). After multivariate Cox regression analysis of the above factors, it could be concluded that the independent risk factors for OS were age, race, T_stage, N_stage, M_stage, grade, CA125, and chemotherapy (Table 2). Independent risk factors of CSS were age, race, marital, T_stage, N_stage, M_stage, grade, CA125, and chemotherapy (Table 3).

**Table 2:** Univariate and Multivariate Analysis of Overall Survival in the Training Group

Variables	Number of patients	Univariate analysis		Multivariate analysis	
		HR (95% CI)	*P* value	HR (95% CI)	*P* value
Age					
12–57	578	Reference		Reference	
58–70	213	1.465 (1.081–1.986)	0.014	1.508 (1.091–2.085)	0.012
71–92	126	3.695 (2.773–4.923)	< 0.001	3.254 (2.292–4.620)	< 0.001
Race					
Black	70	Reference		Reference	
White	742	0.520 (0.360–0.752)	< 0.001	0.510 (0.345–0.754)	< 0.001
Other	105	0.331 (0.191–0.572)	< 0.001	0.500 (0.284–0.883)	0.017
Marital					
Divorced	94	Reference		Reference	
Married	461	0.761 (0.514–1.127)	0.173	0.772 (0.509–1.170)	0.222
Separated	5	1.047 (0.250–4.370)	0.950	0.764 (0.167–3.510)	0.730
Single	265	0.581 (0.375–0.898)	0.015	0.754 (0.475–1.197)	0.232
Unmarried or Domestic Partner	1	1.689*10^(−6)(0-Inf)	0.992	8.143*10^(−6)(0-Inf)	0.998
Widowed	91	1.487 (0.939–2.356)	0.091	0.644 (0.383–1.081)	0.096
Laterality					
unilateral	807	Reference		Reference	
bilateral	110	5.387 (4.135–7.019)	< 0.001	1.258 (0.909–1.741)	0.166
Grade					
I	349	Reference		Reference	
II	405	1.419 (1.051–1.916)	0.023	1.215 (0.892–1.656)	0.218
III	132	3.249 (2.329–4.533)	< 0.001	2.138 (1.471–3.106)	< 0.001
IV	31	2.533 (1.401–4.580)	0.002	2.347 (1.255–4.388)	0.008
T stage					
T1	684	Reference		Reference	
T2	66	3.515 (2.336–5.289)	< 0.001	2.305 (1.465–3.626)	< 0.001
T3	166	10.780 (8.269–14.055)	< 0.001	6.290 (4.290–9.222)	< 0.001
TX	1	2.146*10^(−6)(0-Inf)	0.994	2.473*10^(− 7)(0-Inf)	0.996
N stage					
N0	842	Reference		Reference	
N1	42	5.500 (3.799–7.964)	< 0.001	1.560 (1.030–2.363)	0.036
NX	33	4.657 (3.023–7.172)	< 0.001	1.525 (0.927–2.508)	0.096
M stage					
M0	832	Reference		Reference	
M1	72	10.335 (7.715–13.843)	< 0.001	2.900 (2.046–4.112)	< 0.001
MX	13	5.042 (2.665–9.537)	< 0.001	2.158 (1.045–4.456)	0.038
CA125					
Positive	612	Reference		Reference	
Negative	302	0.273 (0.192–0.389)	< 0.001	0.520 (0.353–0.767)	< 0.001
Borderline	3	1.946*10^(−7)(0-Inf)	0.992	3.821*10^(−7)(0-Inf)	0.993
Surgery					
No	5	Reference		Reference	
Yes	912	0.096 (0.040–0.236)	< 0.001	0.908 (0.342–2.413)	0.847
Radiation					
Yes	6	Reference		Reference	
None/Unknown	911	0.187 (0.077–0.453)	< 0.001	0.998 (0.388–2.562)	0.996
Chemotherapy					
Yes	427	Reference		Reference	
No/Unknown	490	0.616 (0.482–0.788)	< 0.001	1.729 (1.262–2.371)	< 0.001

**Table 3:** Univariate and Multivariate Analysis of Cancer-Specific Survival in the Training Group

Variables	Number of patients	Univariate analysis		Multivariate analysis	
		HR (95% CI)	*P* value	HR (95% CI)	*P* value
Age					
12–57	578	Reference		Reference	
58–70	213	1.374 (0.989–1.908)	0.058	1.390 (0.978–1.975)	0.067
71–92	126	2.848 (2.050–3.956)	< 0.001	2.557 (1.720–3.800)	< 0.001
Race					
Black	70	Reference		Reference	
White	742	0.552 (0.365–0.834)	0.005	0.545 (0.351–0.846)	0.007
Other	105	0.368 (0.202–0.672)	0.001	0.575 (0.308–1.074)	0.083
Marital					
Divorced	94	Reference		Reference	
Married	461	0.712 (0.470–1.080)	0.110	0.740 (0.474–1.156)	0.186
Separated	5	1.143 (0.272–4.800)	0.855	0.707 (0.150–3.333)	0.661
Single	265	0.555 (0.350–0.882)	0.013	0.706 (0.431–1.157)	0.167
Unmarried or Domestic Partner	1	1.596*10^(−6)(0-Inf)	0.993	7.135*10^(−7)(0-Inf)	0.998
Widowed	91	1.081 (0.643–1.817)	0.769	0.502 (0.279–0.905)	0.022
Laterality					
unilateral	807	Reference		Reference	
bilateral	110	6.017 (4.526–7.998)	< 0.001	1.215 (0.863–1.710)	0.265
Grade					
I	349	Reference		Reference	
II	405	1.504 (1.069–2.117)	0.019	1.279 (0.898–1.820)	0.172
III	132	3.809 (2.639–5.499)	< 0.001	2.092 (1.390–3.149)	< 0.001
IV	31	3.312 (1.805–6.078)	< 0.001	2.720 (1.414–5.233)	0.003
T stage					
T1	684	Reference		Reference	
T2	66	4.531 (2.878–7.134)	< 0.001	2.858 (1.729–4.724)	< 0.001
T3	166	14.750 (10.886–19.986)	< 0.001	7.779 (5.104–11.855)	< 0.001
TX	1	2.358*10^(−6)(0-Inf)	0.995	1.483*10^(−7)(0-Inf)	0.997
N stage					
N0	842	Reference		Reference	
N1	42	6.167 (4.167–9.127)	< 0.001	1.525 (0.987–2.357)	0.057
NX	33	5.590 (3.578–8.733)	< 0.001	1.710 (1.019–2.869)	0.042
M stage					
M0	832	Reference		Reference	
M1	72	12.173 (8.930–16.590)	< 0.001	3.058 (2.125–4.401)	< 0.001
MX	13	5.792 (2.950–11.370)	< 0.001	2.651 (1.232–5.701)	0.013
CA125					
Positive	612	Reference		Reference	
Negative	302	0.219 (0.143–0.334)	< 0.001	0.435 (0.272–0.696)	< 0.001
Borderline	3	5.078*10^(−7)(0-Inf)	0.990	3.651*10^(−7)(0-Inf)	0.995
Surgery					
No	5	Reference		Reference	
Yes	912	0.084 (0.034–0.206)	< 0.001	0.738 (0.272–2.004)	0.551
Radiation					
Yes	6	Reference		Reference	
None/Unknown	911	0.156 (0.064–0.380)	< 0.001	1.032 (0.394–2.705)	0.949
Chemotherapy					
Yes	427	Reference		Reference	
No/Unknown	490	0.475 (0.360–0.628)	< 0.001	1.565 (1.098–2.231)	0.013

### Construction of nomograms

**Fig. 3:**
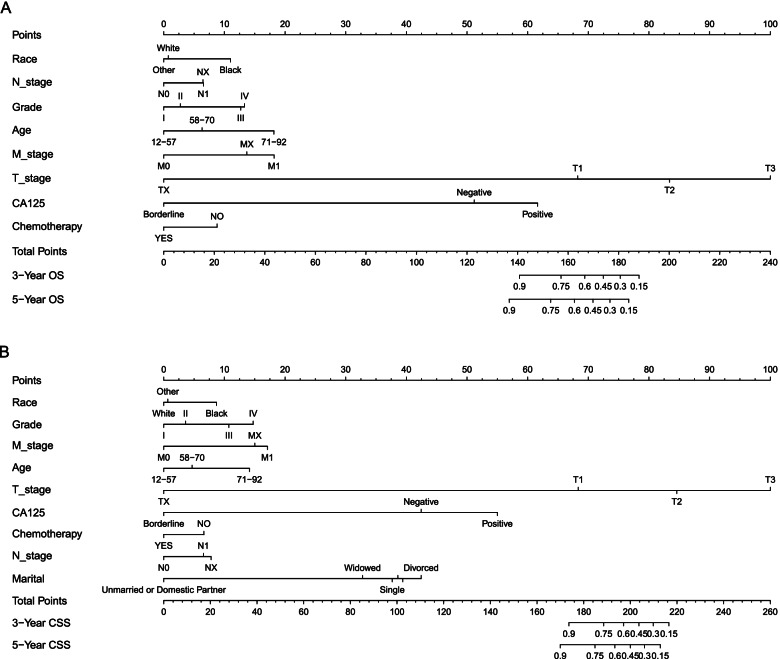
Nomograms for predicting 3-, and 5-year overall survival (OS) and cancer-specific survival (CSS) in ovarian mucinous adenocarcinoma. **A**, 3-, and 5-year overall survival; **B**, 3-, and 5-year cancer-specific survival

The nomogram of OS was constructed according to the independent risk factors (age, race, T_stage, N_stage, M_stage, grade, CA125, and chemotherapy) of OS (Fig. 3). The nomogram of CSS was constructed according to the independent risk factors (age, race, marital, T_stage, N_stage, M_stage, grade, CA125, and chemotherapy) of CSS (Fig. 3).

### Nomogram validation

**Fig. 4:**
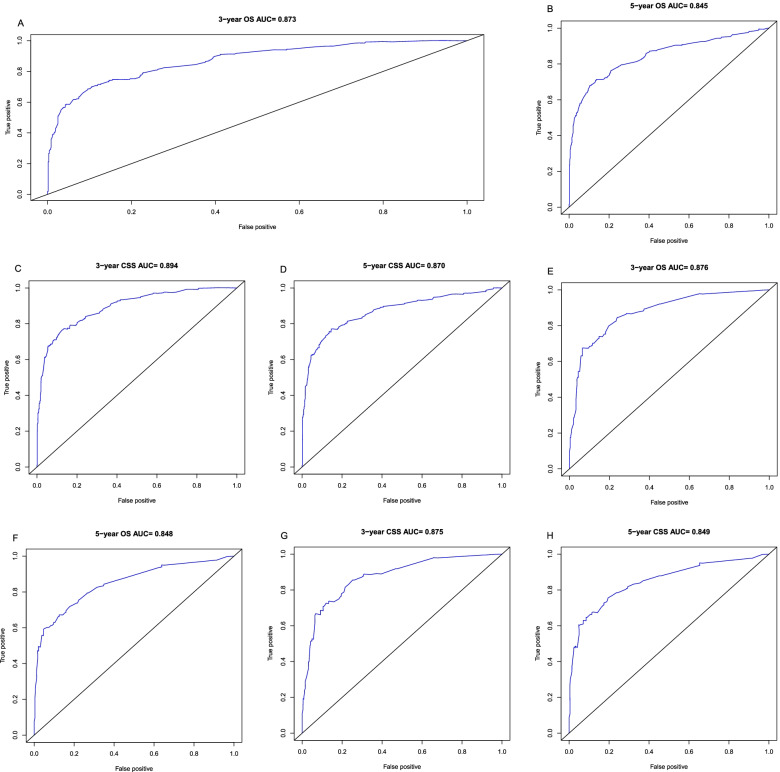
ROC curves in the training (**A**, **B**) and validation (**E**, **F**) groups for 3-, and 5-year overall survival. ROC curves in the training (**C**, **D**) and validation (**G**, **H**) groups for 3-, and 5-year cancer-specific survival. ROC, receiver operating characteristic

**Fig. 5:**
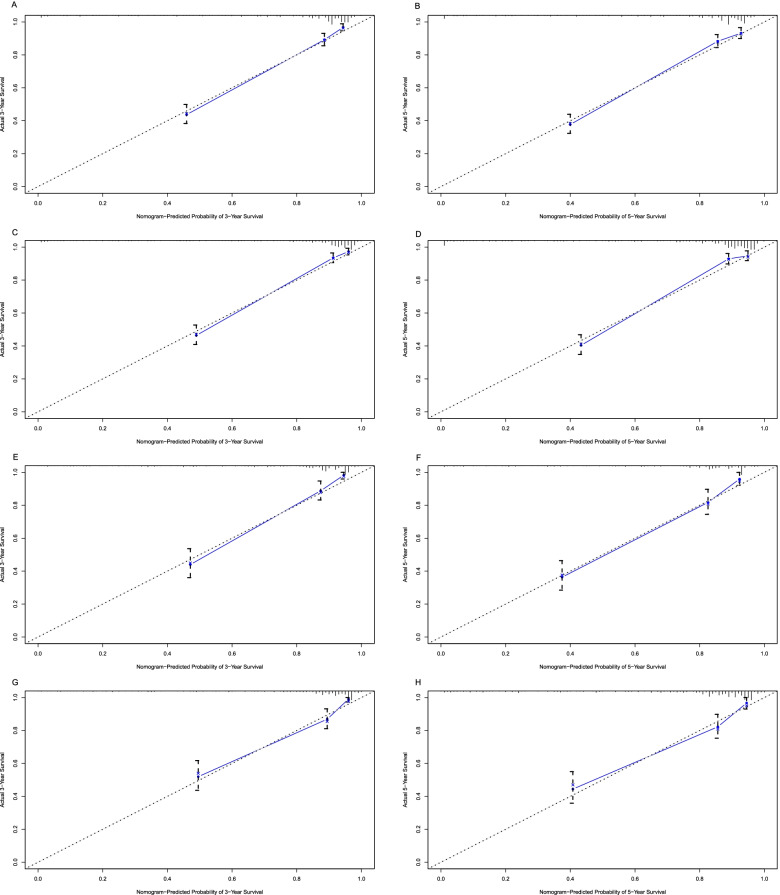
Calibration curves in the training (**A**, **B**) and validation (**E**, **F**) groups for 3-, and 5-year overall survival. Calibration curves in the training (**C**, **D**) and validation (**G**, **H**) groups for 3-, and 5-year cancer-specific survival

In the training group, the C-index of the OS nomogram was 0.845 (95% CI: 0.821–0.869) and the C-index of the CSS nomogram was 0.862 (95%CI: 0.838–0.886). The area under the ROC curves for both 3-year and 5-year OS and CSS nomograms was large in the training group (Fig. 4). The above results indicated that the model had a high discriminative ability. The calibration curves of 3-year and 5-year OS and CSS nomograms in the training group were very close to the reference line, indicating that the accuracy of nomograms was relatively high (Fig. 5).

In addition, we did an internal validation. In the validation group, the C-index of the OS nomogram was 0.843 (95% CI: 0.810–0.876) and the C-index of the CSS nomogram was 0.841 (95%CI: 0.806–0.876). The area under the ROC curves for both 3-year and 5-year OS and CSS nomograms was large in the validation group (Fig. 4). The above results indicated that the model had a high discriminative ability. The calibration curves of 3-year and 5-year OS and CSS nomograms in the validation group were very close to the reference line, indicating that the accuracy of nomograms was relatively high (Fig. 5).

### Kaplan-Meier curves

**Fig. 6:**
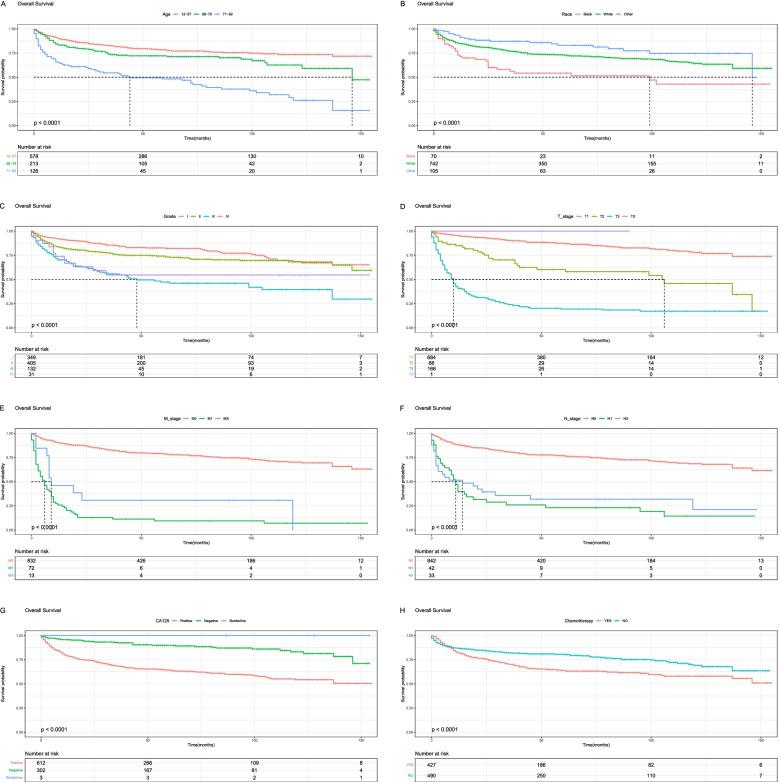
Kaplan-Meier survival curves for overall survival (**A**, **B**, **C**, **D**, **E**, **F**, **G**, **H**) in ovarian mucinous adenocarcinoma patients. **A**, Age; **B**, Race; **C**, Grade; **D**, T_stage; **E**, M_stage; **F**, N_stage; **G**, CA125; **H**, Chemotherapy

**Fig. 7:**
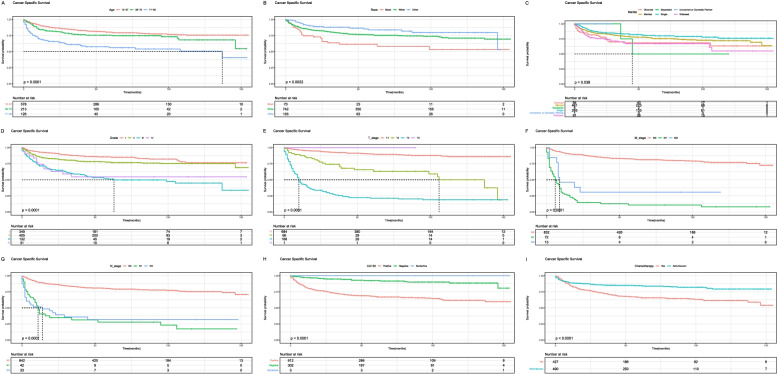
Kaplan-Meier survival curves for cancer-specific survival (**A**, **B**, **C**, **D**, **E**, **F**, **G**, **H**, **I**) in ovarian mucinous adenocarcinoma patients. **A**, Age; **B**, Race; **C**, Marital; **D**, Grade; **E**, T_stage; **F**, M_stage; **G**, N_stage; **H**, CA125; **I**, Chemotherapy

**Fig. 8:**
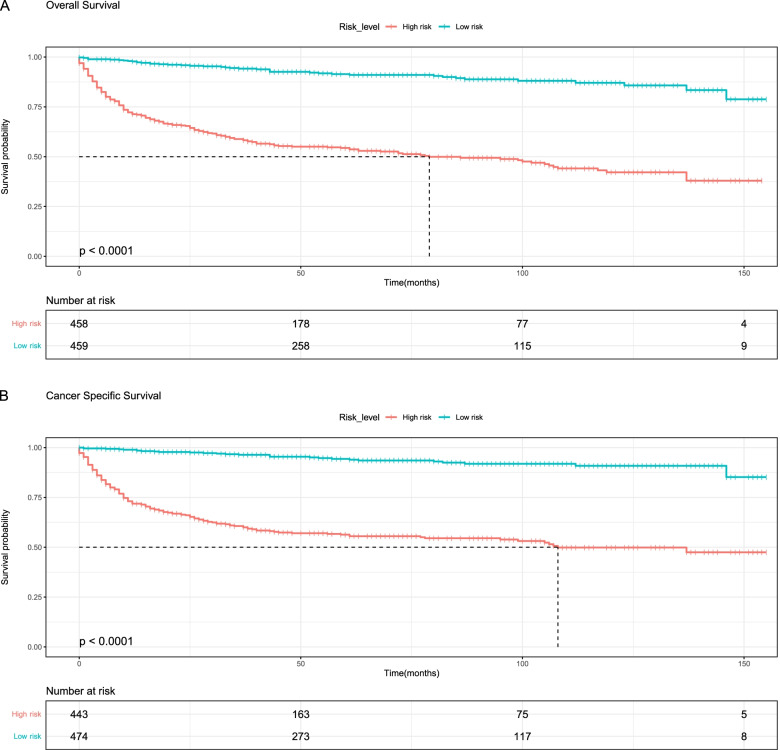
Kaplan-Meier survival curves for the risk-level of overall survival (**A**) and cancer-specific survival (**B**) in ovarian mucinous adenocarcinoma patients

The KM method first calculates the probability that a patient who has survived a certain period of time will survive the next period (the survival probability) and then multiplies the survival probabilities one by one to obtain the survival rate for the corresponding period. Older age (71–92), black race, grade III, IV, advanced T, N, and M stages, CA125 positive, chemotherapy, and high-risk level were associated with lower overall survival of the tumor (Figs. 6 and  8). Older age (71–92), black race, separated, grade III, IV, advanced T, N, and M stages, CA125 positive, chemotherapy, and high-risk level were associated with lower cancer-specific survival of the tumor (Figs. 7 and  8).

## Discussion

Recently, nomograms have been used by clinicians to predict the prognosis of various tumors and formulate individualized treatment plans, such as breast cancer, liver cancer, lung cancer, gastric cancer, and cervical cancer [11–15]. Of course, nomograms also have extensive research in predicting the prognosis of patients with ovarian cancer. Chen et al. [16] selected 1541 patients screened from the SEER database who were initially diagnosed with ovarian clear cell carcinoma (OCCC) during 2010–2016 and randomly divided them into the training group (*n* = 1079) and the validation group (*n* = 462). The 3-year and 5-year OS and CSS nomograms were constructed and validated, with high predictive and clinical value [16]. Wang et al. [17] screened 9001 cases of epithelial ovarian cancer through the SEER database and randomly divided them into the training group (*n* = 6301) and the validation group (*n* = 2700). They constructed and validated 3-year and 5-year OS and CSS nomograms of the disease, which played a role in evaluating prognosis and guiding clinical treatment [17]. Song et al. [18] included 13,403 patients with advanced epithelial ovarian cancer and constructed the early death nomogram of FIGO Stage III and IV epithelial ovarian cancer patients. The internal validation confirmed that the nomogram was highly accurate in predicting premature death and helpful in screening patients with high clinical risk with high practicality [18]. You et al. [19] included 506 eligible patients with postoperative ovarian sex cord-stromal tumor (SCST) from the SEER database. They constructed and validated the OS nomogram, which showed that it was more practical than FIGO staging [19].

As one of the 10 most common cancers in women, the mortality rate of ovarian cancer is increasing [3]. Ovarian mucinous carcinoma is one of the major subtypes of ovarian cancer. A careful understanding of its biological characteristics shows that ovarian mucinous carcinoma is a disease that requires unique treatment. But for a long time, guidelines for ovarian serous carcinoma have been used for the treatment of ovarian mucinous carcinoma [6]. Therefore, it is important to construct a valuable prognostic model to predict the prognosis of ovarian mucinous adenocarcinoma.

In this study, we identified prognostic factors for OS and CSS in the training group by univariate and multivariate Cox regression analysis of the basic information and disease information of patients in the database. Prognostic factors affecting OS were age, race, T_stage, N_stage, M_stage, grade, CA125, chemotherapy. The above factors, together with marital, were prognostic factors for CSS. Based on multivariate Cox regression analysis, we established a prognostic nomogram for OS and CSS in patients with ovarian mucinous adenocarcinoma. In the training group, through the calculation of the C-index, the drawing of the ROC curve and the calibration curve, the results showed that the prognostic model had the higher discriminative ability and higher accuracy. In addition, internal validation was performed by the validation group.

In the nomogram, it could be seen that T_stage played the greatest role in the prognosis prediction, followed by CA125. CA125 is a tumor marker for ovarian cancer. Its sensitivity is related to the stage and histological type of ovarian cancer. It can be used for the early monitoring of disease recurrence and the monitoring of treatment [20]. From the survival curve, we could see that the elderly seemed to have a lower survival rate than the young. Consistent with previous reports, the black race had a lower survival rate than American Indian/AK Native, Asian/Pacific Islander, and white race [21].

In addition, the limitations of this study needed to be noted. Firstly, patients were mainly from the United States, and there was a lack of disease information for patients in other countries, especially Chinese patients with a large population. Secondly, retrospective analysis of the database resulted in selection bias. Finally, the data of surgery, radiation therapy, and chemotherapy in the database only had the results of YES and NO or Unknown and lacked detailed treatment plans.

## Conclusion

In conclusion, this study showed that age, race, T_stage, N_stage, M_stage, grade, CA125, and chemotherapy were independent risk factors for OS in patients with ovarian mucinous adenocarcinoma. In addition, age, race, marital, T_stage, N_stage, M_stage, grade, CA125, and chemotherapy were independent risk factors for CSS. This study constructed and validated 3-year and 5-year prognosis nomograms for OS and CSS in patients with ovarian mucinous adenocarcinoma. Nomograms helped clinicians predict the OS and CSS of patients with ovarian mucinous adenocarcinoma and formulate appropriate treatment plans.

## Data Availability

The datasets analyzed during the current study are available from the publicly available SEER database.
